# Interleukin-22 Signaling in the Regulation of Intestinal Health and Disease

**DOI:** 10.3389/fcell.2015.00085

**Published:** 2016-01-13

**Authors:** Olivia B. Parks, Derek A. Pociask, Zerina Hodzic, Jay K. Kolls, Misty Good

**Affiliations:** ^1^Department of Pediatrics, University of Pittsburgh School of MedicinePittsburgh, PA, USA; ^2^Department of Pediatrics, Richard King Mellon Foundation Institute for Pediatric Research, University of Pittsburgh School of MedicinePittsburgh, PA, USA; ^3^Division of Newborn Medicine, Department of Pediatrics, Children's Hospital of Pittsburgh, University of Pittsburgh School of MedicinePittsburgh, PA, USA

**Keywords:** interleukin-22, gastrointestinal tract, epithelial cells, barrier defense

## Abstract

Interleukin (IL)-22 is a member of the IL-10 family of cytokines that has been extensively studied since its discovery in 2000. This review article aims to describe the cellular sources and signaling pathways of this cytokine as well as the functions of IL-22 in the intestine. In addition, this article describes the roles of IL-22 in the pathogenesis of several gastrointestinal diseases, including inhibition of inflammation and barrier defense against pathogens within the intestine. Since many of the functions of IL-22 in the intestine are incompletely understood, this review is meant to assess our current understanding of the roles of IL-22 and provide new opportunities for inquiry to improve human intestinal health and disease.

## Introduction

Since its discovery in 2000 (Dumoutier et al., 2000a), interleukin-22 (IL-22) has been widely studied for its diverse roles in cell proliferation, tissue regeneration, cellular defense, and inflammation. IL-22 is expressed by inflammatory cells in a number of tissues in the body including the lungs, liver, kidneys, thymus, pancreas, breast, gut, skin, and synovium, reviewed in Dudakov et al. (2015). The focus of this review article is on the distinct roles of IL-22 within the intestine. One of the main functions of IL-22 is to support and maintain the gastrointestinal (GI) epithelial barrier as well as to facilitate barrier defense mechanisms against bacterial pathogens such as *Clostridium difficile* (Hasegawa et al., 2014), *Citrobacter rodentium* (Zheng et al., 2008; Muñoz et al., 2015), and *Toxoplasma gondii* (Muñoz et al., 2015). Studies have shown that regulation of IL-22 is an important component of many diseases including graft-versus-host disease (GVHD) (Munneke et al., 2014), inflammatory bowel disease (IBD), specifically Crohn's disease (Wolk et al., 2007; Schmechel et al., 2008; Souza et al., 2013), ulcerative colitis and the experimental model of ulcerative colitis dextran sodium sulfate (DSS)-induced colitis (Sugimoto et al., 2008) as well as acute polymicrobial sepsis (Weber et al., 2007). The diverse effects of IL-22 against several inflammatory conditions implicate IL-22 as a promising therapeutic target for these types of GI-related illnesses. The purpose of this review article is to describe the distinct roles of IL-22 in the regulation of health and disease in the intestine.

## Cellular sources of IL-22

IL-22 is a member of the IL-10 family and has recently been grouped into a smaller subset of cytokines called the IL-20 subfamily, which is comprised of IL-19, IL-20, IL-22, IL-24, and IL-26 (Ouyang et al., 2011; Rutz et al., 2014). This family was identified based on their cellular targets and ability to recognize similar receptor subunits.

Many different types of immune cells produce IL-22. In lymphoid tissues, αβ and γδ T cells, innate lymphoid cells (ILCs; Cella et al., 2009; Colonna, 2009; Cella et al., 2010; Sonnenberg et al., 2011a; Hanash et al., 2012; Lee et al., 2012; Spits et al., 2013; Korn et al., 2014) and NK T cells (NKTs) all produce IL-22. In addition to the IL-22 produced in lymphoid tissue, macrophages, neutrophils (Zindl et al., 2013; Lee et al., 2015), dendritic cells (Mann et al., 2014), and fibroblasts (Ikeuchi et al., 2005) have also been identified as sources for IL-22; however, these non-lymphoid sources produce less IL-22 relative to the lymphoid sources. Depending on the tissue type, other non-traditional cell types can produce IL-22. For example, in the gastrointestinal tract of mice with experimental colitis, neutrophils become activated and are subsequently capable of expressing IL-22 (Zindl et al., 2013; Lee et al., 2015). In a mouse model of DSS-induced colitis, Zindl et al. demonstrated that depletion of Ly6G/C^+^ neutrophils/dendritic cells using RB6-8C antibody resulted in reduced colonic levels of IL-22, and additionally, these isolated colonic neutrophils were capable of producing IL-22 after IL-23 stimulation (Zindl et al., 2013). In a mouse model of infectious colitis, Lee and colleagues found the CD11b^+^ Ly6C^+^ Ly6G^+^ subset of neutrophils were the main source of IL-22 secretion (Lee et al., 2015). This type of non-traditional expression of IL-22 can be found in patients with diseases outside the GI tract such as rheumatoid arthritis (RA), where fibroblasts have been found to contribute to the production of IL-22 (Ikeuchi et al., 2005). Accordingly, cellular sources of IL-22 are numerous, and the main production sites of IL-22 in lymphoid-derived cells are described below:

### αβ T cells

IL-22 production has been demonstrated by three subtypes of T helper (Th) cells: Th1, Th17, and Th22. The prototypical IFN-γ producing Th1 cells can produce IL-22 without additional growth factors during effector function. However, naive T cells require the presence of transforming growth factor-β (TGF-β) to drive either the formation of Th17 cells, which can then produce IL-22, and/or T regulatory cells (Tregs; Littman and Rudensky, 2010). The cytokine IL-6 drives the expression of retinoic acid-related orphan receptor-γt (RORγt) and IL-23, both of which are critical for Th17 cells to produce IL-22 (Zhou et al., 2007). The regulation of Th17 cells is further controlled by changes in the concentration of TGF-β (Zhou et al., 2008). When the concentration of TGF-β is low, Th17 cells are induced via IL-23-stabilization of RORγt (Zhou et al., 2008). However, when TGF-β is present in high concentrations, expression of the IL-23 receptor is inhibited, reducing the expression of both RORγt and IL-22, while also activating the transcription factor forkhead box P3 (FoxP3; Zhou et al., 2008). This leads to the expansion of Tregs. Thus, high concentration of TGF-β promotes Treg expansion while preventing further expansion of Th17 cells (Zhou et al., 2008). This TGF-β dependent suppression of IL-22 was found to be mediated by c-Maf, a transcription factor that binds to the IL-22 promoter (Rutz et al., 2011). Furthermore, IL-23 can counteract the effects of high concentrations of TGF-β by inducing production of IL-22 (Zhou et al., 2008).

Th22 cells also produce IL-22 and were first identified in the skin as a helper T cell population expressing CCR6, CCR4, and CCR10 (Duhen et al., 2009; Trifari et al., 2009). Th22 cells are classified as a subset of CD4^+^ helper T cells characterized for their production of IL-22, IL-13, and tumor necrosis factor (TNF)-α, but not IL-17, IFN-γ, or IL-4 (Duhen et al., 2009). Th22 cells are further described by their inability to produce T-bet (a transcription factor known to control IFN-γ) and negligible expression of RORγt (a Th17 and IL-22 transcription factor; Duhen et al., 2009). However, the development of CD4+ T cells capable of producing IL-22 is dependent on the aryl hydrocarbon receptor (AhR) and T-bet (Basu et al., 2012). Moreover, a recent study has demonstrated that activation of signal transducer and activator of transcription factor 3 (STAT3) was required for IL-22 production by Th22 cells and was responsible for effective host clearance of infectious colitis (Backert et al., 2014).

### γδ T cells

γδ T cells are found in the intraepithelial lymphocyte (IEL) compartment of the intestine and secrete a variety of cytokines, including a significant amount of IL-22 (Sutton et al., 2009). γδ and αβ T cells share two features in common: (1) expression of RORγt and (2) expression of IL-22 in the presence of IL-23 (Martin et al., 2009; Sutton et al., 2009; Mabuchi et al., 2011; Mielke et al., 2013). Certain types of γδ T cells express Toll-like receptors (TLRs) and can directly interact with specific pathogen products (Martin et al., 2009; Crellin et al., 2010).

γδ T cells have roles in limiting the translocation of pathogens such as *Salmonella typhimurium* and *T. gondii* in the intestinal epithelial tissue (Edelblum et al., 2015). The γδ IELs contribute to the regulation and maintenance of gut homeostasis (Fuell et al., 2015). Studies have shown that mice deficient in γδ IELs (T Cell Receptor δ^−∕−^ mice) have structural differences within their intestine compared to wild-type littermates, including altered intestinal glycosylation and glycan antennae (Fuell et al., 2015). These structural differences can contribute to a lack of mucosal protection in the gut of mice deficient in γδ IELs, indicating γδ IELs and γδ T cells play a role in intestinal host defense (Edelblum et al., 2015; Fuell et al., 2015).

### Innate lymphoid cells (ILCs)

ILCs contribute to the innate and adaptive immunity of the intestine and can be found at mucosal surfaces or in cryptopatches, which are found in the lamina propria of the small intestine beneath the intestinal crypts (Diefenbach, 2012). ILCs are characterized by their lymphoid morphology, absence of cytotoxic capacity, and lack of B or T cell receptors as reviewed in Spits and Cupedo (2012) and Spits et al. (2013). ILCs are a relatively recent addition to the immune cell family and are grouped based on their functional characteristics and expression of cytokines and transcription factors (Spits et al., 2013). Group 1 ILC (ILC1s), defined by their expression of the transcription factor T-bet, produce the Th1 cytokine IFN-γ and are critical to host response of intracellular infections (Klose et al., 2014). Group 2 ILCs (ILC2s) are dependent on the transcription factor GATA3 to produce the Th2 cytokines IL-5 and IL-13 (Hoyler et al., 2012) and are important in helminth infection (Fallon et al., 2006). Group 3 ILC (ILC3s) are defined by their expression of RORγt, production of the Th17 cytokines IL-17 and IL-22, and involvement in defense against extracellular bacterial or fungal infections (Sonnenberg et al., 2011b; Gladiator et al., 2013; Edelblum et al., 2015) as described in Table 1 (Eberl et al., 2015).

**Table 1:** **Description of the different types of innate lymphoid cells (ILCs)**.

**Innate Lymphoid Cells**				
**Cell Type**	**Immunologic function**	**Stimulation**	**Transcription factors**	**Effector cytokines**
ILC1	Intestinal inflammation	IL-12 IL-15 IL-18	T-bet	IFN-γ TNF-α
ILC2	Airway inflammation Helminth infection	IL-25 IL-33 TSLP	RORα GATA3 Bcl11b	IL-4 IL-5 IL-13
ILC3	Intestinal inflammation Gut barrier protection Lymphoid tissue development	IL-23 IL-1β	RORγt AhR	IL-17 IL-22 LT-α_1_β_2_ GM-CSF

Moreover, CD4^+^ T cells can regulate ILC production of IL-22 as well as downstream production of antimicrobial peptides (AMPs) in an IFN-γ dependent manner (Korn et al., 2014). The AMPs evaluated in this study were regenerating islet derived 3 (Reg3) γ and β, members of the C-type lectin family (Gallo and Hooper, 2012), which are induced by IL-22 (Kolls et al., 2008; Zheng et al., 2008). Reg3β has been reported to kill *Escherichia coli* and may be involved in creating a niche of invading pathogens, specifically *S. typhimurium* (Stelter et al., 2011). In contrast, the related protein Reg3γ, which binds to peptidoglycan, has bactericidal activity against Gram-positive bacteria (Cash et al., 2006) and maintains a physical space between luminal bacteria and the epithelium (Vaishnava et al., 2011).

ILC3s can be found in the small and large intestinal mucosa, Peyer's patches and gut-associated lymphoid tissue (GALT; Cella et al., 2014). ILC3s include lymphoid tissue-inducer (LTi) cells and ILC3s that can be activated to express IL-22 by IL-23 through natural cytotoxicity receptor (NCR)^+^ and NCR^−^ ILC3s (Spits and Cupedo, 2012; Spits et al., 2013). In both humans and mice, NCR^+^ ILC3s do not produce IFN-γ, which promote NK cell function, differing these cells from typical NK cells (Colonna, 2009; Cella et al., 2010; Spits et al., 2013). In addition, IL-15 and RORγt are essential for the development and maturation of NCR^+^ ILC3s (Satoh-Takayama et al., 2008; Cella et al., 2009; Luci et al., 2009; Sanos et al., 2009).

### Natural killer T cells

All Natural Killer T (NKT) cells develop and mature in the thymus where they diverge from other cell types at the CD4^+^CD8^+^ double positive thymocyte stage (Mebius et al., 2001; Bendelac et al., 2007; Possot et al., 2011). Before this thymocyte stage, the αβ T cell receptor (TCR) of undifferentiated cells must recombine to bind to a CD1d molecule to progress developmentally (Bendelac et al., 2007). The NKT cell developmental cycle has unique stages of development (Bendelac et al., 2007; Godfrey et al., 2010) based on the NKT cells expression of CD24, CD44, or NK1.1 as well as the transcription factors PLZF, c-Myc, Egr2, RelA, or T-bet (Bendelac et al., 2007; Godfrey et al., 2010). NKT cells that produce IL-22 are also known to express CCR6, IL-23R, RORγt, paralleling γδ T cells, Th17 cells, and ILC3s (Bendelac et al., 2007; Godfrey et al., 2010). In addition, NKT cells produce IL-22 in the presence of IL-23 (Rachitskaya et al., 2008; Doisne et al., 2011; Moreira-Teixeira et al., 2011; Paget et al., 2012). Accordingly, NKT cells are similar to ILCs and γδ T cells in that these cell types can produce IL-22 without transcriptional activator interferon regulatory factor 4 (IRF4) signaling, whereas αβ T cells require IRF4 signaling to initiate IL-22 expression (Raifer et al., 2012). However, IL-22-producing NKT cells have been found to require interaction from TCR-CD1d to induce production of IL-22 (Doisne et al., 2011). Many details surrounding NKT cells that produce IL-22 are not well understood. A study has suggested that a stage during NKT cell development called “stage 0” (CD24^+^CD44^lo^NK1.1^−^) is crucial to the maturation of these cells and is hypothesized to be controlled by RORγt (Benlagha et al., 2005); however, further studies are needed to completely understand the role of NKT cells in IL-22 production.

## Positive and negative regulators of IL-22

IL-22 is mainly produced by Th17 cells, ILCs, γδ T cells, and NKT cells (Ouyang et al., 2011). Th17 cells produce IL-22 in response to IL-6 and TNF-α in the setting of inflammation and trauma (Liang et al., 2006; Zhang et al., 2011). However, IL-6 can independently initiate the expression of IL-22 (Liang et al., 2006; Zheng et al., 2007). *Il-22* gene expression, initially found only in the thymus and brain (Dumoutier et al., 2000a,b; Sabat et al., 2014), has been discovered in the gastrointestinal tract, liver, lung, skin, pancreas, and spleen (Sabat et al., 2014). IL-22 expression and production is positively and negatively regulated by several molecules including IL-23, IL-7, the Notch signaling pathway, the AhR, IL-22 binding protein (IL-22BP), IL-25, and IL-1β. Our continued study of these molecules can aid in the understanding IL-22, particularly in the setting of gastrointestinal disease.

### Interleukin-23

IL-23 is one of the main inducers of the expression and production of IL-22 (Kastelein et al., 2007). During differentiation of Th17 cells, IL-23 enhances IL-22 expression, which leads to increased expression of the IL-23 Receptor (IL-23R). This results in enhanced interaction between IL-23 and its receptor, and consequently, increased IL-22 production. Activated dendritic cells (DCs) and macrophages, in response to microbial stimulation, are important sources of IL-23 (Langrish et al., 2004). IL-23 production by DCs is regulated by the lymphotoxin-β receptor (LTβR; Tumanov et al., 2011). IL-23 production by activated DCs via LTBR indirectly results in increased IL-22 by RORyt+ ILCs (Tumanov et al., 2011).

However, there are other cellular sources of IL-23. DSS-induced epithelial injury results in activation of LTβR signaling, which promotes intestinal mucosal healing by triggering production of IL-23 from intestinal epithelial cells (Macho-Fernandez et al., 2015). This ultimately results in increased IL-22 production by RORyt+ ILC3s, particularly CCR6+Tbet- CD4- and CD4+ LTi cells (Macho-Fernandez et al., 2015).

### Interleukin-7

IL-7, a critical cytokine for the maintenance, development, and proliferation of αβ and γδ T cells, also acts to positively regulate the production of IL-22 (Cella et al., 2010). It is unlikely that the cytokine IL-7 directly regulates the expression of IL-22; however, IL-7 is necessary for the stable expression of RORγt, a transcription factor important in IL-22 expression (Vonarbourg et al., 2010). RORγt expression regulates the differentiation of cells that produce IL-22 to achieve the optimal conditions for the production of IL-22 (Nurieva et al., 2007; Qiu et al., 2012). These observations would suggest that IL-7 acts in an expansion role to promote the expression of IL-22 by many types of cells. However, IL-7 is not *directly* required for functional IL-22 cytokine to be produced in tissues (Peschon et al., 1994; von Freeden-Jeffry et al., 1995; Nurieva et al., 2007; Vonarbourg et al., 2010; Qiu et al., 2012).

### Notch

Notch-induced stimulation of CD4^+^ T cells increases the production of IL-22 within the intestine, which is important for epithelial cell proliferation and differentiation (Murano et al., 2014). Overexpression of Hes1, a Notch target gene, enhanced IL-22-induced STAT3 expression in a human intestinal epithelial cell line (Murano et al., 2014). However, in Notch-deficient mice, IL-22 signaling and production was eliminated (Alam et al., 2010; Murano et al., 2014). Lee et al. demonstrated that the Notch receptor was induced by the AhR (Lee et al., 2012), which promotes the development of ILCs and Th17 cells, ultimately leading to increased IL-22 production.

### Aryl hydrocarbon receptor (AhR)

The AhR induces IL-22 in one of two ways. AhR can either (1) directly regulate IL-22 gene expression and cytokine production or (2) regulate the production and development of ILC3 and Th17 cells (Veldhoen et al., 2008; Lee et al., 2012; Qiu et al., 2012). AhR is found in the cytoplasm complexed with heat shock protein 90 (Hsp90; Esser et al., 2009), and once activated, the AhR complex can translocate to the nucleus where AhR can act as a transcription factor (Tsuji et al., 2014). Various ligands, physical stress, cyclic AMP, and calcium (Ca^2+^) can all activate AhR. It is thought that AhR ligands derived from gut microbiota are not required for the development of ILCs (Veldhoen et al., 2008; Esser et al., 2009; Qiu et al., 2012). However, these ligands are thought to be an essential component to initiate the transcription of IL-22 (Denison and Nagy, 2003; Oesch-Bartlomowicz et al., 2005; McMillan and Bradfield, 2007; Nguyen and Bradfield, 2008; Puga et al., 2009; Lee et al., 2012; Zelante et al., 2013; Lowe et al., 2014).

### Interleukin-1β

IL-1β is an influential cytokine, capable of activating NKT cells, ILC3s, and Th17 cells to produce IL-22 (Sutton et al., 2009; Doisne et al., 2011; Paget et al., 2012; Chen et al., 2013; Lee et al., 2013; Monteiro et al., 2013). Macrophages, DCs, neutrophils, B and T cells, endothelial cells, and epithelial cells all produce IL-1β, making this ligand a diverse molecule in cellular pathways (Sims and Smith, 2010). IL-1β production sustains expression of IL-22 *in vitro*, promotes the expansion of NK cells, specifically, human stage three immature NK cells, in conjunction with IL-15 from secondary lymphoid tissue DCs, and inhibits these NK cells from differentiating into IFN-γ-producing cells (Sutton et al., 2009). Of note, in IL-1 receptor^hi^ immature NK cells, IL-1β must be *constantly* present in order for continual expression of IL-22. This is in direct contrast to IL-23, which only needs to be present to *initiate* the IL-22 signaling pathway so that IL-22 production continues in the absence of IL-23 (Hughes et al., 2010).

### Negative regulation of IL-22

There are several molecules that negatively regulate IL-22 expression. IL-22 Binding Protein (IL-22BP, also known as IL-22RA2) is a soluble receptor that is able to regulate IL-22 bioactivity, which has a 1000 times higher binding affinity for IL-22 compared to the IL-22 receptor (IL-22RA1) complex (Weiss et al., 2004; Wolk et al., 2007). Epithelial expression of IL-25 is able to repress IL-22 production by RORyt+ ILCs (Weiss et al., 2004; Wolk et al., 2007). Transforming growth factor-β (TGF-β) has several functions, specifically with respect to Th17 cells, RORγt, IL-23R, and IL-22 (Mangan et al., 2006; Zhou et al., 2007; Morishima et al., 2009). TGF-β is essential for the differentiation of Th17 cells and influences RORγt and IL-23R expression in various tissues (Morishima et al., 2009). In IL-22 signaling, TGF-β acts in a dose-dependent fashion to regulate the expression of IL-22 (Zheng et al., 2007; Volpe et al., 2009; Rutz et al., 2011; Penel-Sotirakis et al., 2012). However, the cytokine IL-23 is able to overcome the effects of TGF-β on IL-22, thereby increasing production of IL-22 (Volpe et al., 2009; Rutz et al., 2011). Furthermore, both the transcription factor c-Maf (an inhibitor of TGF-β, IL-22, IL-27) and the inducible costimulator (ICOS) pathways can influence IL-22 production (Bauquet et al., 2009; Paulos et al., 2010; Rutz et al., 2011). A recently described and poorly understood cytokine member of the IL-1 family, IL-38, can also regulate IL-22 production. At low concentrations, IL-38 can prevent the production of IL-22, while at high concentrations, IL-38 promotes the production of IL-22 (Tortola et al., 2012; van de Veerdonk et al., 2012). However, further studies are required to determine the signaling pathways involved with IL-38 and their biologic effects.

### IL-22 binding protein

IL-22RA2, alternatively called IL-22BP, has been found throughout the body. It is a soluble secreted receptor with a binding structure similar to the membrane bound IL-22RA1 (Dumoutier et al., 2001; Wu et al., 2008). This structural homology allows IL-22BP to bind to IL-22, thereby inhibiting the binding of IL-22 to its receptor complex and consequently preventing IL-22 signaling (Wu et al., 2008). Interestingly, it has been observed that IL-22BP levels decrease with significant increases in IL-22 (Weiss et al., 2004; Wolk et al., 2007). IL-22BP levels increase only after persistently high levels of IL-22, indicating that IL-22BP has a regulatory role after the initial effects of the elevated IL-22 levels have been established (Wolk et al., 2007; Sugimoto et al., 2008; Huber et al., 2012). In the intestine, IL-22BP is highly expressed in colonic dendritic cells (Huber et al., 2012). When tissue damage in the intestine is detected, the NLRP3 or NLRP6 inflammasomes down-regulate IL-22BP via activation of IL-18 (Huber et al., 2012). Additionally, Martin et al. discovered a subset of conventional dendritic cells, lamina propria CD103^+^CD11b^+^ DCs, in both lymphoid and non-lymphoid tissues as a significant source of IL-22BP (Martin et al., 2014). Additionally, eosinophils within the human intestine were also identified as an important source of IL-22BP (Martin et al., 2015).

## IL-22 receptor and signaling

The IL-22 receptor is a type 2 cytokine receptor comprised of the heterodimeric complex with IL-22RA1 and IL-10R2 (IL-10Rβ; Kotenko et al., 1997, 2001; Xie et al., 2000; Dumoutier et al., 2000c; Li et al., 2004). While IL-10R2 is constitutively expressed in cells throughout the body, IL-22RA1 is expressed almost exclusively in epithelial tissues (Wolk et al., 2004). Due to this specificity of IL-22RA1, it is hypothesized that this receptor has a defining role in facilitating the innate immunity of epithelial cells (Zheng et al., 2007, 2008). Interestingly, IL-22 has been shown to have no affinity for IL-10R2 and rather a very high affinity to bind to IL-22RA1 (Logsdon et al., 2002, 2004; Li et al., 2004; Wolk et al., 2004; Jones et al., 2008; Yoon et al., 2010). Binding of IL-22 to IL-22RA1 increases its affinity for IL-10R2 (Logsdon et al., 2002; Li et al., 2004; Logsdon et al., 2004; Wolk et al., 2004; Jones et al., 2008; Yoon et al., 2010), suggesting a stepwise process in the binding (Bleicher et al., 2008).

IL-22 signals through the IL-22 receptor complex, leading to activation of Janus kinase 1 (Jak1) and non-receptor protein tyrosine kinase 2 (Tyk2; Lejeune et al., 2002). This primarily leads to tyrosine residue phosphorylation of STAT3; however, STAT1 and STAT5 have also been shown to be activated by IL-22 (Lejeune et al., 2002). STAT3 activation in the intestinal epithelium is responsible for immune homeostasis as well as wound healing in an IL-22 dependent manner (Pickert et al., 2009). Several additional pathways are involved in IL-22 signaling, including Mitogen Activation Protein Kinase (MAPK), Akt (Sekikawa et al., 2010), and p38 pathways (Andoh et al., 2005).

## IL-22 regulation of health and disease: gastrointestinal tract

IL-22 has several roles in the gastrointestinal tract, including tissue regeneration and cell proliferation, defense against pathogens, as well as maintenance and protection of the intestinal barrier. Expression of IL-22 has been identified in the many tissues including the upper GI tract, the oral cavity, salivary glands, tonsils, stomach, and esophagus (Cella et al., 2009; Cupedo et al., 2009; Hughes et al., 2009; Ciccia et al., 2012; Delsing et al., 2012; Kato-Kogoe et al., 2012; Naher et al., 2012; Zhuang et al., 2012). In these tissues, IL-22 induces the production of defensins, Reg family molecules, and S100 proteins—all of which are innate antimicrobial molecules (Wolk et al., 2004, 2006; Liang et al., 2006; Zheng et al., 2008; Pickert et al., 2009; Sanos et al., 2011; Sonnenberg et al., 2012). These antimicrobials assist in providing gut barrier protection against pathogens (Sugimoto et al., 2008). During colitis, IL-22 is responsible for regulating mucin production from goblet cells, which constitutes the protective mucous layer that lines the intestinal epithelium (Sugimoto et al., 2008). However, further studies are needed to determine whether the intestinal mucin production is directly or indirectly related to goblet cells influenced by IL-22 expression.

Recent studies have demonstrated that patients with IBD, specifically Crohn's disease or ulcerative colitis have increased IL-22 expression in the colonic tissue (Hanash et al., 2012). Wolk et al. found systemically elevated IL-22 in Crohn's disease and intestinal elevation of IL-22 in a mouse model of colitis (Wolk et al., 2007). In both circumstances, LPS-binding protein (LBP) was also found to be upregulated in the blood (Wolk et al., 2007). Moreover, administration of IL-22 to healthy mice resulted in increased LBP at concentrations capable of neutralizing LPS, and consequently, inflammation. This suggests that IL-22 can act as an anti-inflammatory molecule against LPS and may be a novel therapeutic agent in IBD patients (Witte et al., 2010; Sonnenberg et al., 2011a; Sabat et al., 2014).

In patients with IBD, there is significantly decreased expression of AhR in intestinal tissue, and in response to AhR agonist, 6-formylindolo(3, 2-b) carbazole (Ficz), isolated intestinal lamina propria mononuclear cells in this patient population resulted in increased IL-22 (Monteleone et al., 2011). Moreover, in multiple experimental mouse models of colitis, administration of Ficz subsequently caused IL-22 induction, whereas administration of an AhR antagonist resulted in more severe colitis with decreased IL-22 production (Monteleone et al., 2011).

IL-22 has a critical role in antibacterial immunity and host defense in the intestine. IL-22 is important in the clearance of the mouse pathogen *C. rodentium*, which causes infectious colitis that mimics *E. coli* infection in humans (Zheng et al., 2008; Sonnenberg et al., 2011b). Furthermore, infectious colitis with *C. rodentium* can be suppressed with ILC production of IL-22 (Qiu et al., 2012) in an AhR and microbiota-dependent manner (Qiu et al., 2013). This is in accordance with the observation that IL-22 is elevated in the colon in response to infection by *C. difficile* or *C. rodentium* (Sonnenberg et al., 2011b). This suppression against *C. rodentium* infection is mediated by ILCs, which are the main sources of IL-22 in the intestine (Hanash et al., 2012). ILCs have also been associated as a source of IL-22 for defense against DSS-induced colitis and GI graft-versus-host disease (GVHD; Hanash et al., 2012). ILCs have also been implicated in the pathogenesis of IBD, as they are present within the inflamed intestinal tissue of patients. Studies by Hepworth et al. demonstrate that ILCs regulate CD4^+^ T cell responses to commensal bacteria within the intestine (Hepworth et al., 2013). Future research is necessary to understand how ILCs manipulate the adaptive immunity to protect against diseases such as IBD. Manipulation of ILCs or the cytokines they produce may provide therapeutic targets in the future for patients with IBD.

IL-22 can also provide pro-inflammatory responses within the intestine. In some cases, such as those involving colonic subepithelial myofibroblasts, increasing IL-22 levels will cause inflammation and hyperproliferation, resulting in negative effects on tissues and the production of pro-inflammatory molecules like IL-1, IL-6, IL-8, IL-11, G-CSF, and GM-CSF (Andoh et al., 2005). Increased IL-22 levels can also recruit pathologic effector cells to the inflamed tissue site, most noticeably in autoimmune diseases in other tissues (Pan et al., 2013).

### Host defense against bacterial pathogens in intestine

One important mechanism of host defenses in the intestine against bacterial pathogens are the presence of tight junctions that maintain the integrity of the intestinal epithelium, thereby preventing bacterial translocation (Macdonald and Monteleone, 2005), and notably, IL-22 is capable of maintaining these tight junctions (Kim et al., 2012). In addition to the mucin-secreting goblet cells within the intestine, Paneth cells are able to maintain mucosal integrity against pathogens by releasing AMPs, which help contain microorganisms within the GI tract (Takahashi et al., 2001). Zheng and colleagues investigated the role of IL-22 in protecting colonic tissue from infectious colitis with *C. rodentium* (Zheng et al., 2008), paralleling infection by enterohemorrhagic *E. coli* (EHEC), and enteropathogenic *E. coli* (EPEC) in humans (Mead and Griffin, 1998). EHEC and EPEC both cause infectious diarrhea and carry a high morbidity and mortality, most noticeably in infants and children in developing countries (Zheng et al., 2008). The murine pathogen *C. rodentium* provides an experimental model of infectious colitis in mice. Zheng et al. compared IL-22-deficient mice to wild-type mice infected with *C. rodentium* and found that IL-22-deficient mice had 80–100% mortality during the second week post-infection (Zheng et al., 2008). Wild-type mice in response to infection by *C. rodentium* initially experienced weight loss, but were able to make a full recovery from the infection after ~6 days (Zheng et al., 2008). Histological analysis of these two groups revealed increased colonic mucosal hyperplasia and increased inflammation in the submucosal tissue in IL-22-deficient mice compared to the wild-type mice (Zheng et al., 2008). Moreover, the site of infection by *C. rodentium* differed in the two groups of mice (Zheng et al., 2008). In the IL-22-deficient mice, bacteria were found deep in the colonic tissue, whereas bacteria in wild-type mice were limited to the superficial layer of the epithelial tissue (Zheng et al., 2008). Furthermore, the authors concluded that IL-22 is the only indispensable cytokine necessary to ensure host defense against *C. rodentium* during the early stages of the infection as opposed to IL-17A, IL-17F, IL-19, IL-20, and IL-24 (Zheng et al., 2008). If IL-22 is absent during initial infection by *C. rodentium*, IL-22 does not provide adequate protection against the bacteria (Zheng et al., 2008). IL-22 knock-out mice infected with *C. rodentium* that were administered IL-22 several days after infection had a high mortality rate compared to infected mice administered IL-22 at the beginning of infection (Zheng et al., 2008). This data is a strong indication that IL-22 is an important cytokine in protecting intestinal epithelial tissue from bacterial pathogens and may provide a therapeutic opportunity against EHEC and EPEC infectious colitis in humans (Zheng et al., 2008).

The microbiota within the intestine contain pathobionts, which are commensal bacteria that can become virulent when homeostasis is disrupted (Hasegawa et al., 2014). For example, when intestinal epithelial tissue is infected with the pathogen *C. difficile*, pathobionts are capable of translocating to other tissues, thereby spreading the infection (Hasegawa et al., 2014). However, IL-22 can control the elimination of enterobacterial pathobionts by facilitating the binding of C3 of the complement pathway to bacteria (Hasegawa et al., 2014). Since little is understood about immune mechanisms in defense against these pathobionts, the discovery of the role IL-22 plays in controlling the pathobionts is important.

### IL-22 and fucosylation in the intestine

**Figure 1:**
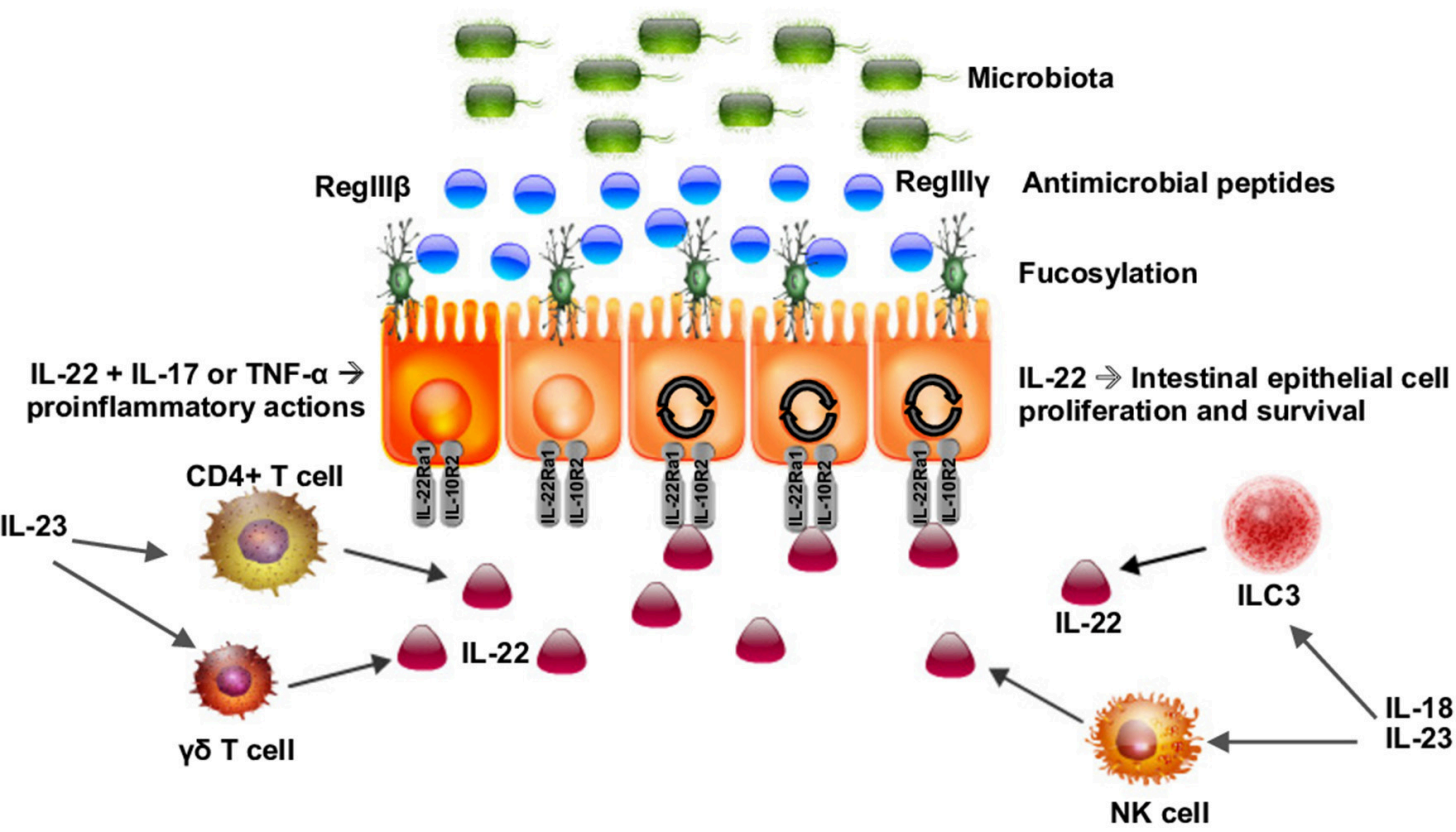
**Schematic of IL-22 as a key regulator of the interaction of the microbiota with the intestinal epithelium**. IL-22 is produced by CD4+ and γδ T cells as well as type 3 innate lymphoid cells (ILC3s). IL-22 acts on intestinal epithelial cells inducing fucosylation and the release of antimicrobial peptides (AMPs), regulating the microbiota, and maintaining gut barrier homeostasis. IL-22 can act synergistically with IL-17 and TNF-α to activate a proinflammatory response to certain pathogens.

Recently, the IL-22RA1 receptor has also been shown to mediate protection against *C. rodentium* infection in the intestine (Pham et al., 2014). Mice deficient in IL-22RA1 had increased epithelial bacterial translocation of *Enterococcus faecalis* compared to littermate controls. When fucosylated oligosaccharides were administered to the *C. rodentium*-infected IL-22RA1-deficient mice, infection was attenuated and the bacterial diversity of commensals was restored, demonstrating that IL-22RA1 mediates antimicrobial activities and intestinal fucosylation as in Figure 1 and (Pham et al., 2014). Intestinal epithelial cell fucosylation is catalyzed by fucosyltransferase 2 (Fut2) and is a symbiotic mechanism of host-microbiota interaction, as many bacteria utilize epithelial fucose as a source of dietary carbohydrate (Pacheco et al., 2012; Goto et al., 2014). Pickard et al. demonstrated that Fut2 was expressed by IL-22-stimulated intestinal epithelial cells in the intestine and that fucosylation of the intestinal epithelium occurred in response to Toll-like receptor ligand exposure (Pickard et al., 2014).

Segmented filamentous bacteria (SFB) are an intestinal commensal bacteria in mice that can induce IL-22 expression (Ivanov et al., 2009). Fucosylation in the ileum of SFB-colonized mice has been shown to be dependent on IL-22 and the TNF family member lymphotoxin α (LTα; Goto et al., 2014). IL-22 and LTα are produced by ILC3s to mediate intestinal fucosylation demonstrating that ILC3s may control the intestinal commensals by this mechanism (Goto et al., 2014).

### Graft-versus-host disease (GVHD)

Graft versus host disease can occur after donor T cells are activated against antigens from the recipient and subsequently attack various organs such as the intestinal tract (Hanash et al., 2012). Multiple investigators have shown that IL-22 derived from the recipient in the liver and GI tract have been shown to reduce mortality and tissue pathology, whereas donor-derived IL-22 has the opposite effect, increasing mortality and inflammation in target tissues (Hanash et al., 2012; Couturier et al., 2013; Zhao et al., 2013, 2014). Furthermore, recipient-derived IL-22 is produced by ILCs, while donor-derived IL-22 is produced by donor T cells (Hanash et al., 2012). It is thought that the opposing effects may be due to several different mechanisms including the target cells and distinct localizations of donor T cells and recipient ILCs in tissues (Hanash et al., 2012). The potential benefits of IL-22 in GVHD may also be limited since recipient IL-22^+^ ILCs can be removed by alloreactive donor T cells (Hanash et al., 2012). There is a clinical trial in progress to assess the safety and tolerability of recombinant human IL-22 IgG2-Fc (F-652) in combination with systemic corticosteroids for the treatment of acute gastrointestinal GVHD in hematopoietic stem cell transplantation recipients (ClinicalTrials.gov identifier NCT02406651; Generon Corporation Memorial and Sloan Kettering Cancer, 2016). It has further been noted that there may be a parallel between IL-22 deficiency in GVHD and the condition autoimmune polyendocrinopathy-candidiasis-ectodermal dystrophy (APECED), in which antibodies have the ability to neutralize cytokines such as IL-22 (Kärner et al., 2013; Laakso et al., 2014). As IL-22 levels decrease in patients with APECED, they can develop an increased susceptibility to candida infections (Kärner et al., 2013; Laakso et al., 2014).

IL-22 stimulates the production of molecules from cells within the intestinal epithelium (Peterson and Artis, 2014). For example, molecules like Reg3 and the defensins are thought to be regulated by IL-22 (Kolls et al., 2008; Salzman and Bevins, 2013). However, Paneth cells can produce Reg3 and defensins with limited evidence that IL-22 is solely responsible for this effect (Kolls et al., 2008; Salzman and Bevins, 2013). In addition, a common pathological finding in GVHD is a decrease in the number of Paneth cells (Eriguchi et al., 2012). Therefore, further research is required to understand the role of IL-22 in the regulation and production of these molecules in order to understand pathogenesis of diseases of the GI tract such as GVHD.

### Maintenance of the GI epithelial barrier

Cross talk between IL-22, ILC3s, and the microbiota within the intestine contributes to the regulation and maintenance of the intestinal epithelial barrier by IL-22 (Sonnenberg et al., 2012). ILC-derived IL-22 has been found to be essential for preventing systemic inflammation, specifically containing species like *Alcaligenes*, a genus of Gram-negative bacteria residing within the mesenteric lymph nodes and Peyer's patches (Sonnenberg et al., 2012). Mice that are deficient in IL-22 display very few epithelial tissue perturbations (Zheng et al., 2008; Sonnenberg et al., 2011b, 2012). However, when these mice are exposed to *C. rodentium*, they develop severe colitis (Zheng et al., 2008; Sonnenberg et al., 2011b). Studies have shown that colitis can be reversed by the introduction of IL-22 (Sonnenberg et al., 2012). In a similar study, mice deficient in ILC3s were found to also be very susceptible to DSS-induced colitis (Sawa et al., 2011). This suggests that ILC3-derived IL-22 plays a protective role in the epithelial tissue against pathogens (Sawa et al., 2011).

The study by Backert et al. (2014) on infectious colitis revealed that the activation of STAT3 in CD4+ cells is necessary for the expression of IL-22 to facilitate host defense against *C. rodentium* infection. Mice deficient in STAT3 in CD4+ cells mice exhibited no change in the initial course of their infection during the innate lymphoid cell-dependent phase (Backert et al., 2014). However, during the lymphocyte-dependent phase of infection, these mice displayed an augmented distribution of the bacteria as well as significant defects in the intestinal epithelial barrier (Backert et al., 2014). Specifically, the lamina propria was notably significantly deficient in IL-22-producing CD4^+^ lymphocytes (Backert et al., 2014). This observation suggests that both Th17 and Th22 cells are dependent on STAT3 activation to promote the production of IL-22 (Backert et al., 2014). In mice with active STAT3, the intestinal epithelial barrier was intact and functional, successfully protecting against enteropathogenic bacteria (Backert et al., 2014). These results contribute to our understanding of the mechanisms underlying the role IL-22 in maintenance of the gastrointestinal epithelial integrity.

In bacterial infections caused by *C. rodentium* and *T. gondii*, Munoz and colleagues found that intestinal epithelial cell production of IL-18 is mediated by IL-22 in these two models of intestinal inflammation (Muñoz et al., 2015). IL-18 is required for ILCs to express IL-22, and IL-22 was shown to increase the expression of IL-18 mRNA in the gastrointestinal tissue (Muñoz et al., 2015). In IL-22-deficient mice, there was a reduction in Th1 cells promoting IL-18 expression (Muñoz et al., 2015). Specifically in *C. rodentium* infection, both IL-22 and IL-18 together contribute to barrier defense against the infection (Muñoz et al., 2015). In *T. gondii* infection, IL-18 is required for IL-22 production in the ileum (Muñoz et al., 2015). This study demonstrated the mutual regulation between IL-18 and IL-22 in defense against intestinal infections (Muñoz et al., 2015). These studies contribute to the understanding of the regulation of IL-22 in maintaining gastrointestinal integrity against bacterial pathogens.

In addition to bacterial pathogens, IL-22 can also assist in controlling viral infections. ILC3 production of IL-22 was found to upregulate IFN-λ by intestinal epithelial cells and act synergistically to control rotavirus infection (Hernández et al., 2015), a common diarrheal infection in childhood. The effect of IL-22 on controlling rotavirus replication was dependent on IFN- λR signaling and STAT1 activation and independent of STAT3 (Hernández et al., 2015). These data provide evidence that IL-22 may also provide effective clearance of other GI related viral illness.

### The function of IL-22 in secondary lymphoid tissue

The role that IL-22 plays in secondary lymphoid tissue and mucosa-associated lymphoid tissues (MALTs) including lymph nodes, cryptopatches, isolated lymphoid follicles (ILFs), and Peyer's patches has not been fully elucidated. A study demonstrated that stimulation of Peyer's patches with IL-23 induced NKp46+ NK cells to produce IL-22 (Yoshida et al., 1999; Sun et al., 2000; Eberl and Littman, 2003; Cupedo et al., 2004; Finke, 2005; Tsuji et al., 2008). Furthermore, ILF development is dependent on LTi cells, which produce IL-17, IL-22, and LTα_1_β_2_ (Ota et al., 2011). The study by Ota et al. demonstrated that the LTα_1_β_2_ pathway has an important role in IL-22 production during infection with *C. rodentium* (Ota et al., 2011). When the LTα_1_β_2_ pathway was blocked, colonic IL-22 expression was significantly decreased and exogenous IL-22 administration attenuated colonic damage related to *C. rodentium* infection (Ota et al., 2011). This suggests that IL-22 may be an essential contributor to the maintenance of intestinal lymphoid tissue in the presence of inflammation. Moreover, further studies revealed that the AhR drives IL-22 production, and AhR-deficient mice were not protected against *C. rodentium* infection due in part to a defect in IL-22 production in the lamina propria and Peyer's patches (Zheng et al., 2007). AhR-deficient mice were also found to have a lack of cryptopatches and mature ILFs without affecting formation of Peyer's patches (Zheng et al., 2007). Future studies are warranted to determine whether IL-22 directly or indirectly participates in the maintenance of the GI tract lymphoid tissue and host defense against pathogens in the presence of an infection.

### Acute polymicrobial sepsis

Weber et al. investigated the role of IL-22 in polymicrobial peritonitis (Weber et al., 2007). The authors infected mice that had been administered recombinant IL-22BP in the form of an Fcγ2a fusion protein (Weber et al., 2007). Mice were treated with IL-22BP-Fc (an IL-22 antagonist; Xie et al., 2000; Dumoutier et al., 2001; Nagalakshmi et al., 2004) 4 hours before polymicrobial septic peritonitis (Weber et al., 2007). Analysis of these mice revealed that there was a significant accumulation of neutrophils and mononuclear phagocytes in conjunction with reduced bacterial load at the direct point of infection (Weber et al., 2007). Additionally, the liver and kidneys of these mice experienced increased bacterial clearance, and kidney injury was ameliorated (Weber et al., 2007). As sepsis progressed in polymicrobial peritonitis, activation of IL-22RA1 and induction of IL-22 produced a pro-inflammatory response that exacerbated the infection (Weber et al., 2007). Bacterial spread and organ failure, which are consequences of polymicrobial peritonitis, appeared to increase due to the role of IL-22 in the progression of sepsis (Weber et al., 2007). They found that both IL-22 and IL-10 were expressed in the presence of sepsis as a consequence of polymicrobial peritonitis (Weber et al., 2007). However, IL-22 was predominantly expressed in the spleen and kidneys, while IL-10 was more widespread in several organs including the spleen, kidney, and liver (Weber et al., 2007). These findings suggest IL-22 influences the production of cytokines and affects the ability of the tissues to provide antibacterial host defense mechanisms in the presence of acute sepsis and peritonitis (Weber et al., 2007). Accordingly, IL-22 has a significant role in acute infection. Furthermore, the IL-22BP derived from Fcγ2a fusion protein can serve as an antagonist to the effects of IL-22 during acute sepsis, and suggests a potential therapeutic intervention for polymicrobial peritonitis (Weber et al., 2007).

## Conclusions

In conclusion, IL-22 plays an important role in the pathogenesis of many intestinal diseases. IL-22 promotes epithelial wound healing and proliferation of several cell types and in various tissues. Furthermore, the studies described in this review highlight IL-22 as a potential and promising therapeutic target for many gastrointestinal diseases. Extensive research on IL-22 is necessary to fully describe and explain the differences in the ability of this cytokine to provide pro- or anti-inflammatory responses in particular tissues and disease states. A deeper understanding of the regulation and function of IL-22 provides a potential opportunity for the development of novel preventative or therapeutic approaches to many diseases.

## Funding

DP is supported by R01HL122760. JK is supported by R01HL062052 and R37HL079142. MG is supported by K08DK101608 from the National Institutes of Health and the Children's Hospital of Pittsburgh of the UPMC Health System.

### Conflict of interest statement

The authors declare that the research was conducted in the absence of any commercial or financial relationships that could be construed as a potential conflict of interest.
